# A multi-network integration approach for measuring disease similarity based on ncRNA regulation and heterogeneous information

**DOI:** 10.1186/s12859-022-04613-1

**Published:** 2022-03-07

**Authors:** Ningyi Zhang, Tianyi Zang

**Affiliations:** Department of Computer Science and Technology, Harbin Institute of Technology, Harbin, China

**Keywords:** Non-coding RNA, Disease similarity, Semantic association, Gene functional network

## Abstract

**Background:**

Measuring similarity between complex diseases has significant implications for revealing the pathogenesis of diseases and development in the domain of biomedicine. It has been consentaneous that functional associations between disease-related genes and semantic associations can be applied to calculate disease similarity. Currently, more and more studies have demonstrated the profound involvement of non-coding RNA in the regulation of genome organization and gene expression. Thus, taking ncRNA into account can be useful in measuring disease similarities. However, existing methods ignore the regulation functions of ncRNA in biological process. In this study, we proposed a novel deep-learning method to deduce disease similarity.

**Results:**

In this article, we proposed a novel method, ImpAESim, a framework integrating multiple networks embedding to learn compact feature representations and disease similarity calculation. We first utilize three different disease-related information networks to build up a heterogeneous network, after a network diffusion process, RWR, a compact feature learning model composed of classic Auto Encoder (AE) and improved AE model is proposed to extract constraints and low-dimensional feature representations. We finally obtain an accurate and low-dimensional feature representation of diseases, then we employed the cosine distance as the measurement of disease similarity.

**Conclusion:**

ImpAESim focuses on extracting a low-dimensional vector representation of features based on ncRNA regulation, and gene–gene interaction network. Our method can significantly reduce the calculation bias resulted from the sparse disease associations which are derived from semantic associations.

## Background

Human complex diseases are often related with each other through shared causes or pathology. Knowledge of how various diseases are related can facilitate deepening the understanding of their etiology and pathogenesis [1, 2]. Studying the relationships can make contributions to predict disease causing genes [3, 4], inferring miRNA function associations [5, 6], and identifying novel therapeutic drugs for diseases [7, 8]. Various aspects including pathogenesis and phenotypes can be exploited to calculate the similarity of pairwise diseases. Current methods for measuring disease similarity can be classified as semantic-based [5, 9] and functional-based [10–12]. Semantic-based methods are widely used for measuring similarity between diseases-associated ontological terms, such as Gene Ontology [13] and human phenotype ontology (HPO) [14] in biomedical and bioinformatics domain. Semantic association between diseases are documented in the ontology such as Disease Ontology (DO) [9]. For measuring similarity of semantic associations, Resnik’s method calculates disease similarity based on the information content (IC) of the most informative common ancestor (MICA) between two terms, Wang et al.’s method calculate similarity between terms considering multiple common ancestors [15]. It has been successfully employed in measuring disease similarity between medical subject headings (MeSH) terms and inferring microRNA function network [16]. Le et al. constructed disease similarity network based on semantic similarity measures on phenotype ontology database and integrated them with several kinds of gene/protein networks [17]. MultiSourcDSim proposed by Deng et al. compute the similarity between diseases by integrating multiple biological datasets including gene-disease associations, GO biological process-disease associations and symptom-disease associations [18].

Function-based methods for calculating similarity of terms incorporate genome information. Mathur and Dinakarpandian presented a process-similarity based (PSB) method by involving the associations based on Gene Ontology [13]. Cheng et al. utilized gene interactions in the comprehensive gene functional network to calculate disease similarity (SemFunSim) [19]. In contrast with aforementioned methods which ignore that genes could also be associated based on intermediate nodes in the gene functional network, InfDisSim presented by Hu et al. models the information flow to the network in order that the entire network could be fully utilized [20]. Keller et al. revealed hidden relationships between diseases based on common associated genes as well as genes associated with a common set of diseases by investigating formal concepts [21]. Carson et al. assumed that if a gene or gene sets is related to only one pair of diseases, the similarity between these two diseases would be higher than that of a pair of diseases sharing gene associations with many other diseases [22]. However, it is worth noticing that many of these methods calculate disease similarity based on a single metric or a single data source, which could lead to a biased conclusion lacking of comprehensive assessment. Moreover, non-coding RNA have been demonstrated that they play a major part in many significant biological process, but existing methods have not taken this into account.

Non-coding RNAs have been considered as key regulators of gene expression, genome stability and defense against foreign genetic elements. The majority of the human genome transcripts are non-coding RNAs, in particular, miRNAs and lncRNAs [23, 24], which are involved in a plethora of cellular processes including either cis- or trans-regulation of protein-coding genes and alternative splicing. In this work, we developed a novel method, called ImpAESim, to calculate disease similarity with taking these non-coding RNAs into account by integrating multiple disease information networks. Many existing methods have been proposed to ensemble multiple networks into one network, such as kernel-based [25], Bayesian inference-based [26], weighted averaging or summing-based approaches [27], deep learning models [28, 29], and network representation learning (NRL) methods [30], these aforementioned methods fuse different networks into one integrated network and extract feature representations. However, they may induce information loss in the process of summarizing different networks into one. To solve this problem, multi-network embedding methods have been proposed, such as Mashup [31] which captured low-dimensional feature representations of genes based on multiple networks by utilizing a matrix factorization-based approach. However, matrix factorization-based approaches are a kind of linear and limited approach, it is difficult to capture complex and high-dimensional non-linear structure in integrated networks.

To address above problems, we proposed a novel method, named ImpAESim (disease similarity calculation based on an improved Auto-Encoder model), ImpAESim not only integrates diverse information from heterogeneous data sources (e.g., disease-gene associations, lncRNA-gene associations, miRNA-gene associations) but also copes with the noisy and high-dimensional nature of large-scale biological data by utilizing an improved Auto-Encoder (AE) model to learn low-dimensional but informative vector representations of disease features. Then by measuring the distance between pairwise diseases we finally obtained the disease similarity. To this end, ImpAESim is not only a novel method to calculate disease similarity but also provide a new aspect to enrich human understanding of the heterogeneity and relevance of diseases.

## Results

### Effectiveness

**Fig. 1:**
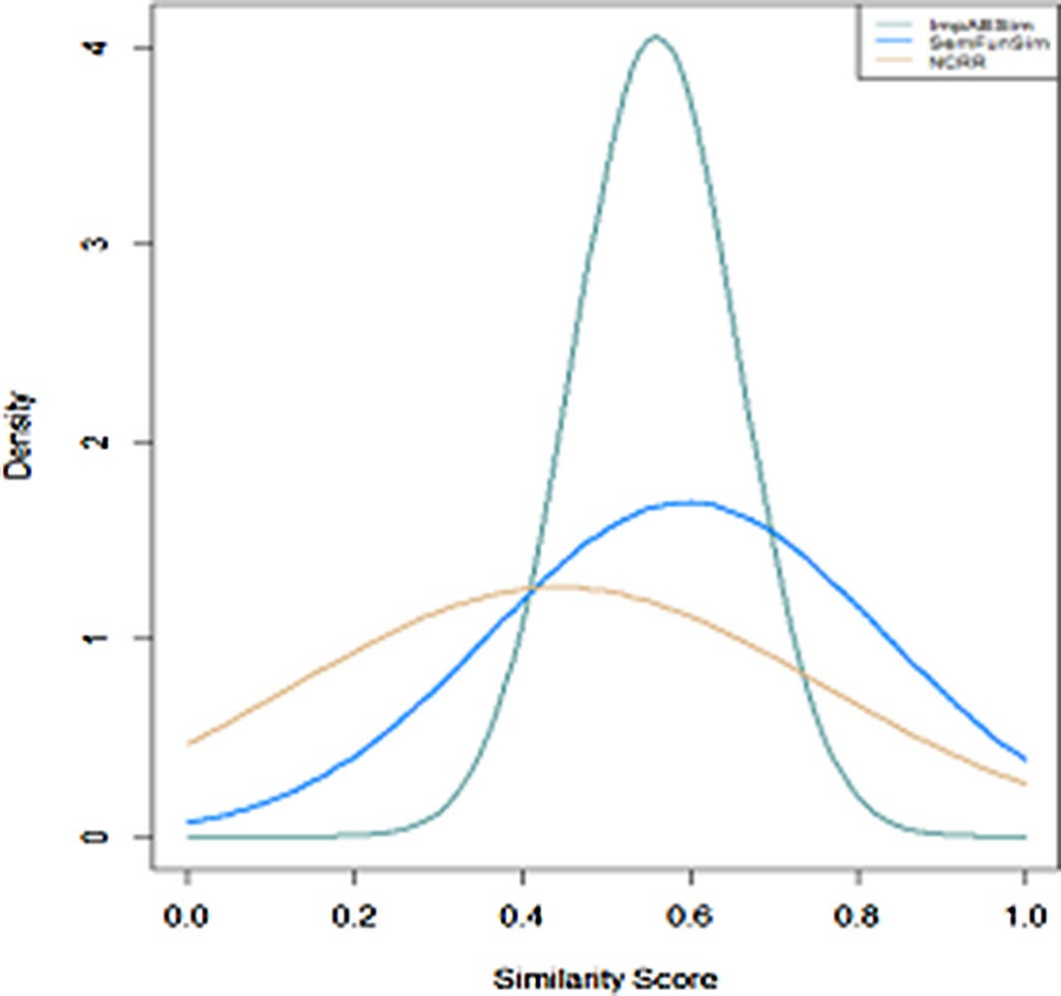
Density curves of three disease similarity methods

Figure 1 shows the distribution of similarity scores calculated by ImpAESim, SemFunSim and NCRR. After normalization, the similarity score of 1,390,206 disease pairs of 1,405,326 range from 0.3 to 0.8. In order to further analyze the performance of the proposed method, ImpAESim was compared with disease similarity methods SemFunSim and NCRR. During the experiment, the parameters of these methods are selected according to the original paper. To clearly study the density curves, the disease pairs with similarity score under 0.2 and 0.3 are omitted for SemFunSim and NCRR, respectively. SemFunSim and NCRR both are similarity methods utilizing disease terms and ‘is_A’ relationships from Disease Ontology database. From this aspect, similarity score of many disease pairs may be 0 because they have no relationships according to semantic terms. Therefore, in the figure of distribution density curves of SemFunSim and NCRR, they spread wider than the density curve of ImpAESim, this indicates that the results of SemFunSim and NCRR are loosely structured, which is not beneficial to study the relationships of different diseases. To further test the efficiency of ImpAESim. We randomly selected five diseases from the disease set as the query diseases, and a list comprising of a top-5 most similar diseases to each query disease generated by ImpAESim. The results were recorded in Table 1. Take Hyperbilirubinemia for example, ImpAESim has discovered that porphyria was similar or related to it with the given disease set. Many studies on these two diseases have revealed their close relationship, such as hyperbilirubinemia is observed in erythropoietic porphyrias [32]. For coronary artery disease and familial benign chronic pemphigus, it has been detected that mutations in exons of ATP2C1 gene in the patients of familial benign chronic pemphigus [33], Nassa et al. found that ATP2C1 may induce coronary artery disease [34].

**Table 1:** Top-5 similar diseases for 5 query disease

Query	Top-5 associated diseases	Score
Coronary artery disease	FBCP	0.7908
	SEMD	0.7886
	RT-syndrome^a^	0.7879
	PMDS	0.7796
	MODY	0.7521
Genetic obesity	GSD	0.8131
	VSD	0.7925
	PC1/3 deficiency	0.7166
	LEP deficiency	0.6885
	SMS	0.6517
Hyperbilirubinemia	SLC anemia	0.8598
	Porphyria	0.8396
	DJS	0.8376
	Rotor syndrome	0.7561
	ILL	0.7461
Neuroblastoma	macrocolon	0.8136
	ADHD	0.8054
	HD	0.78821
	SCDO	0.78649
	PNPO deficiency	0.74891
Growth hormone deficiency	IGH deficiency	0.65643
	CPHD	0.65444
	Hypopituitarism	0.62002
	CRMO	0.61325
	CAGSSS	0.57399

^a^Rubinstein–Taybi syndrome

### Case study

Three diseases Mitochondrial complex I deficiency, Mitochondrial complex II deficiency, and Mitochondrial complex III deficiency were selected as the targets and analyzed for further evaluation of the effectiveness of our method. Besides, two non-related diseases Precocious puberty and Celiac disease were selected as the contrasts. Table 2 presents the similarity score of each disease pair measured by our method.

**Table 2:** Simialrity Score of 3 disease pairs measured by ImpAESim

Group	Disease-pair	Score
Target	MCDI^a^, MCDII^b^	0.67298
	MCDI, MCDIII^c^	0.66728
	MCDIII, MCDII	0.78042
Contrast1	MCDI, Precocious puberty	0.23109
	MCDII, Precocious puberty	0.30141
	MCDIII, Precocious puberty	0.33356
Contrast2	MCDI, Celiac disease	0.38606
	MCDI, Celiac disease	0.3198
	MCDI, Celiac disease	0.36077

^a^Mitochondrial complex I deficiency

^b^Mitochondrial complex II deficiency

^c^Mitochondrial complex III deficiency

It is known that Mitochondrial complex I deficiency, Mitochondrial complex II deficiency, and Mitochondrial complex III deficiency are all clinically belong to certain congenital disorder of metabolism. According to the international statistical classification of disease and related health problems (ICD-11) released by world health organization, these three diseases are all found to be the children of the term Inborn errors of energy metabolism. Moreover, in KEGG pathway maps, they all corresponds to pathway Oxidative phosphorylation (hsa00190), while Mitochondrial complex II deficiency also corresponds to pathway Citrate cycle (TCA cycle, hsa00020) and Mitochondrial complex III deficiency corresponds to pathway Cardiac muscle contraction (hsa04260). As a contrast, precocious puberty is a type of endocrine disease and celiac disease is a kind of digestive system disease. Both of them have not been found to have any associations with the above three targeted diseases.

## Discussion

Existing methods for calculating disease similarity most focus on semantic associations, disease gene associations, and gene functional networks. These methods mostly depend on ontology, which are not reliable due to the differences between disease terms from various databases. However, non-coding RNAs such as lncRNAs and miRNAs are also very important in understanding the mechanism of complex diseases. In this article, we proposed a novel method, ImpAESim, a framework integrating multiple networks embedding to learn compact feature representations and disease similarity calculation. We first utilized three different disease-related information networks to build up a heterogeneous network, after a network diffusion process, RWR, a compact feature learning model composed of classic AE and improved AE is proposed to extract constraints and low-dimensional feature representations. We finally obtained an accurate and low-dimensional feature representation of diseases, then we employed the cosine distance as the measurement of disease similarity. This work may facilitate relevant studies and can be further improved to attain more accurate results.

## Conclusions

Complex diseases are not simply caused by a single gene, single mRNA transcript or single protein but the effect of their functional-collaborations. Measuring similarity between complex diseases has significant implications for revealing the etiology and pathogenesis of diseases and further research in the development of biomedicine, which can also support identifying potential therapeutic drugs for diseases.

It has been consentaneous that functional associations between disease-related genes and semantic associations can be applied to calculate disease similarity. Currently, more and more studies have demonstrated a profound involvement of non-coding RNA in the regulation of genome organization and gene expression. Non-coding RNA seem to operate at several biological levels such as epigenetic processes that control differentiation and development. Thus, taking non-coding RNA into account can be useful in measuring disease similarities.

The results of ImpAESim lead us to a further direction in complex disease research. In this paper, we focus on the problem of computing disease similarity with disease associated non-coding RNAs and compact feature learning, which can improve the accuracy of similarity calculation by solving the problem raised by sparse disease associations.

## Methods

### Work frame

**Fig. 2:**
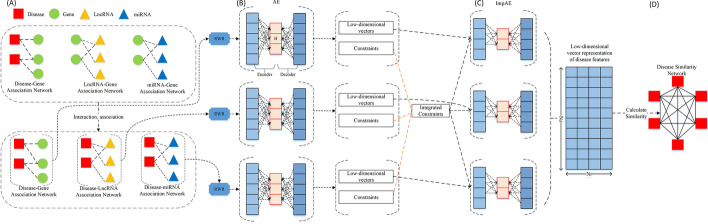
The pipeline of ImpAESim algorithm. This framework mainly contains two parts, multi-network embedding to obtain a compact low-dimensional vector feature representation to describe the topological properties for each disease and disease similarity calculation based on distance measurement. First we integrate three disease-related information sources to construct three input networks (**A**), then we run RWR to learn global topological properties of the networks. The output of RWR is fed to the classic Auto-Encoder (**B**) to calculate the constraints and obtain low-dimensional vectors of hidden layer. Then the low-dimensional vectors and constraints are fed to the ImpAE (**C**) to obtain the low-dimensional representation of disease features after concatenating the hidden vectors. Finally the combined representations of diseases can be utilized to measure disease similarity by calculating a cosine distance (**D**)

ImpAESim contains two main parts, (1) multiple networks embedding based on random walk with restart (RWR) and Auto-Encoder, (2) disease similarity calculation. In the network embedding process, we first utilized a network diffusion algorithm (RWR) to capture single network topological information and transform it into feature representations of each disease node. However, due to the noisy and high-dimensional character of biological network, we need further apply the Auto-Encoder (AE), a deep-learning model to learn the features with extracted constraints from different input networks. Then the low-dimensional feature representations for each disease are obtained by integrating the constraints and hidden vector of the Auto-Encoder by the proposed improved AE model (ImpAE). Intuitively, the low-dimensional vector representations encode the association information and topological context of each disease in the heterogeneous network. After obtaining the low-dimensional feature representation of diseases, ImpAESim calculates the cosine distance as the measurement of disease similarity. The workflow of ImpAESim is presented in Fig. 2.

### Data collection

The heterogeneous network input to ImpAESim is constructed based on the following known biomedical entities: disease-gene associations, gene-miRNA associations, gene-lncRNA associations. Thus, a total of four types of nodes and four types of edges, representing different diseases-related information, were collected from the public databases and used to construct the heterogeneous network for the following work. We collected disease-related genes from KEGG DISEASE Database, it contains 6310 gene-disease associations between 5236 genes and 1907 diseases. We obtained genes regulated by lncRNAs from LncRNA2Target [35]. LncRNA2Target is a database to provide a comprehensive resource of lncRNA-target relationships inferred from low-throughput experiments or lncRNA knockdown or overexpression experiments followed by microarray/RNA-seq. Associations between miRNA target genes and miRNAs are downloaded from miRDB database [36]. All the targets in miRDB were predicted by MirTarget, which was developed by analyzing thousands of miRNA-target interactions from high-throughput sequencing experiments. In addition, we excluded those isolated nodes which means we only keep the nodes with at least one edge in the network. Finally, we got 1677 diseases, 2963 genes, 50 lncRNAs and 2728 miRNAs, with 4647 associations between diseases and genes, 170,226 associations between diseases and miRNAs, 5640 associations between diseases and lncRNAs 660,401 associations between genes and miRNAs, 10,154 associations between genes and lncRNAs.

### ImpAESim algorithm

#### Network embedding and compact feature learning

**Fig. 3:**
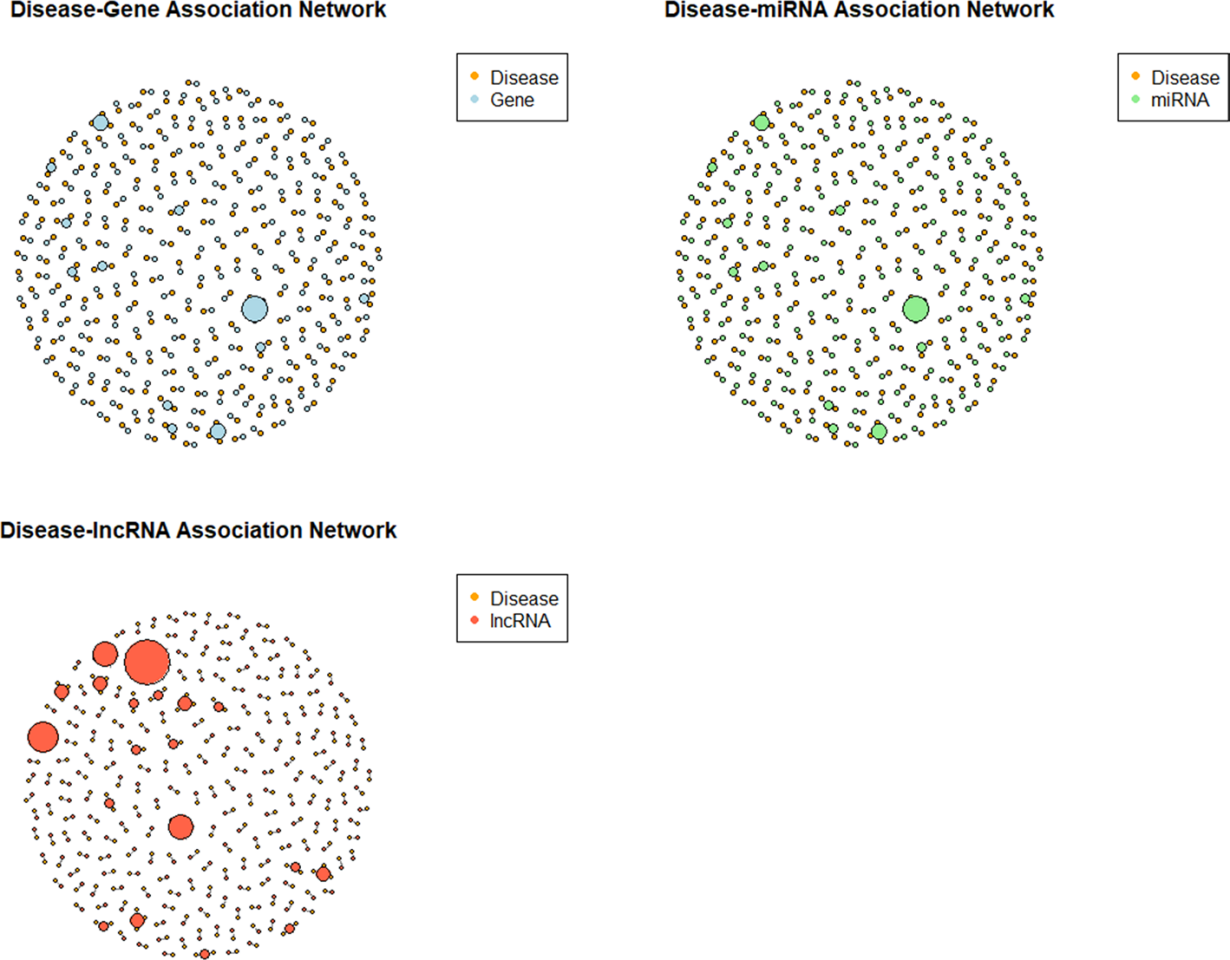
Different disease-related information networks feeding to RWR process

To compile various curated disease-related information, we constructed a heterogeneous network which includes three diverse networks (disease-gene association network, disease-lncRNA association network, and disease-miRNA association network, as shown in Fig. 3). First we utilized a network diffusion algorithm, random walk with restart, RWR, to capture the topological information of each network and transform it into feature representations of nodes. RWR introduces a pre-defined restart probability at the initial node for every iteration, which can take into consideration of global topological connectivity patterns within the network to fully exploit the latent direct or indirect relations between nodes. Formally, let A denote the weighted adjacency matrix of a molecular interaction network with n diseases. Matrix B is defined as a transition matrix, in which B_*i,j*_ denotes the probability of a transition from node i to node j, which means,

1 $$B_{i,j} = \frac{{A_{i,j} }}{{\mathop \sum \nolimits_{{j^{\prime}}} A_{{i,j^{\prime}}} }}$$

Then, $p_{i}^{t}$ denotes an n-dimensional feature vector of disease i in which each element stores the probability of a node being visited from node i after t iterations in the random walk process. Thus, the RWR process from node i can be defined as:

2 $$p_{i}^{t + 1} = \left( {1 - u_{r} } \right)Bp_{i}^{t} + u_{r} e_{i}$$

where $e_{i}$ indicates the n-dimensional standard basis vector with $e_{i} \left( i \right) = 1$ and $e_{i} \left( j \right) = 0,{ }\forall i \ne j$, and $u_{r}$ denotes the pre-defined restart probability, after a range of iterating process, we can obtain a stationary distribution $p_{i}^{\infty }$ of RWR process.

Then each of the disease information network obtained after RWR process is fed into the original autoencoder. For a pair of disease nodes i and j, we first utilized Pearson correlation coefficient (PCC) to measure the pairwise similarity between them. Let *x*_*i*_ and *x*_*j*_ denotes the feature vectors of node i and j, the PCC of them can be indicated as:

3 $$\begin{aligned} PCC_{{x_{i} ,x_{j} }} & = \frac{{cov\left( {x_{i} ,x_{j} } \right)}}{{\sigma_{{x_{i} }} \cdot \sigma_{{x_{j} }} }} \\ & = \frac{{\sum (x_{im} - \overline{{x_{i} }} )\sum \left( {x_{jm} - \overline{{x_{j} }} } \right)}}{{\sqrt {\mathop \sum \nolimits_{m = 1}^{n} \left( {x_{im} - \overline{{x_{i} }} } \right)^{2} } \cdot \sqrt {\mathop \sum \nolimits_{m = 1}^{n} \left( {x_{jm} - \overline{{x_{j} }} } \right)^{2} } }} \\ \end{aligned}$$

#### Constraints extraction using ImpAE

After obtaining the distances between all pairs of disease nodes from each network, two thresholds for positive-link and negative-link are set to extract both constraints. Let $T_{1} { }$ and $T_{2}$ denote the positive-link threshold and negative-link threshold, respectively. Thus, if the PCC value of a pair of nodes is larger than $T_{1}$, the pair is considered as a positive-link constraint, and if the PCC value is smaller than $T_{2}$, the pair is considered as a negative-link constraint.

By compiling the both kinds of constraints, we can get a set of positive-link constraints which means each pair of nodes in it is strongly associated and a set of negative-link constraints which means each pair of nodes in it are unrelated. As a result, the size of constraint sets is much smaller than that of the original network. Thus, the constraints can be considered as the correlation of different networks for the following work.

#### Constraints integration using ImpAE

AE is a typical unsupervised deep learning model which aims to learn a new encoding representation of input data with a superiority in dimensionality reduction. In this work, we proposed a novel optimized autoencoder model named ImpAE to learn the low-dimensional feature representation based on integrating correlations of different networks. The input of ImpAE includes low-dimension feature vector and constraints obtained from former layer. Because the constraints are derived from different networks, before feeding into the ImpAE, we need to take the intersection of the constraints in case that they may conflict with each other. As shown in Fig. 1, the output of RWR process is first input to original AE, then the output of original AE is fed to ImpAE.

Original autoencoder model is composed of two parts, encoder and decoder. The ‘encoder’ operation converts the original high-dimensional data to low-dimensional feature vectors, and the ‘decoder’ operation recovers the input data from the low-dimensional feature vectors. The output low-dimensional feature vectors are considered as a compact representation of the original input data. Let $x_{{\text{i}}}$ be the *i*th input vector indicating the node representation of the network, and *f*, *g* be the activation functions of the hidden layer and the output layer, respectively. Then the output representation of hidden layer and output layer can be indicated as follows,

4 $$h_{i} = f\left( {Wx_{i} + b} \right)$$

5 $$y_{i} = g\left( {W^{\prime } h_{i} + d} \right)$$

where Ω = (W, b, $W^{\prime}$, d) are the parameters, *f* and *g* are activate functions, here we chose the sigmoid function. Then the optimization goal is to minimize the reconstruction error between the reconstructed vector $y_{{\text{i}}}$ and the input vector $x_{{\text{i}}}$,

6 $${\text{arg}}\mathop {\min }\limits_{{\omega \in {\Omega }}} \mathop \sum \limits_{i = 1}^{n} \left\| {y_{{\text{i}}} - x_{{\text{i}}} } \right\|_{2}^{2}$$

After obtaining the constraints from the original AE, the low-dimensional feature vectors and constraints are fed into ImpAE to learn the new representation of the feature vectors. Intuitively, if node i node j are a pair of positive-link constraint, the distance between them should be smaller after encoding. On the contrary, if they are a pair of negative-link constraint, the distance between them should be larger after encoding. Let $h_{{\text{i}}}$ and *h*_*j*_ be the output of encoding operator in the AE, which represent the low-dimensional feature vectors of disease i and j. let $x_{{\text{i}}}$ and *x*_*j*_ denotes the original feature vectors of disease i and j which are the input of the encoding operator. Let d($h_{{\text{i}}} ,$ *h*_*j*_) and d($x_{{\text{i}}} ,$ *x*_*j*_) indicate the error score between disease i and j in the encoding space and original space, respectively. From the hypothesis we mentioned above, d($h_{{\text{i}}} ,h_{{\text{j}}}$) should be smaller than d($x_{{\text{i}}} ,$ *x*_*j*_) if node i and j are a pair of positive-link constraints, d(($h_{{\text{i}}} ,$ *h*_*j*_) should be larger than d($x_{i} ,$ *x*_*j*_) if node i and j are a pair of negative-link constraints. Hence, we add a penalty on the loss function if disease pair (i, j) is a positive-link constraint and we add a reward on the loss function if disease pair (i, j) is a negative-link constraint. Therefore, the loss function for modeling constraints is defined as follows:

7 $$\begin{aligned} {\text{Loss}}_{c} & = \gamma_{1} \mathop \sum \limits_{{\left( {i,j} \right) \in P}} d\left( {h_{{\text{i}}} ,h_{{\text{j}}} } \right) - \mathop \sum \limits_{{\left( {i,j} \right) \in N}} d\left( {h_{{\text{i}}} ,h_{{\text{j}}} } \right) \\ & = \gamma_{1} \mathop \sum \limits_{i,j = 1}^{n} P_{i,j} \left\| {h_{{\text{i}}} ,h_{{\text{j}}} } \right\|_{2}^{2} - \gamma_{2} \mathop \sum \limits_{i,j = 1}^{n} N_{i,j} \left\| {h_{{\text{i}}} ,h_{{\text{j}}} } \right\|_{2}^{2} \\ \end{aligned}$$

where P, N indicates the constraints sets of positive-link constraints and negative-link constraints, respectively. $\gamma_{1}$*,* $\gamma_{2}$ are the weight coefficients restraining the influence of penalty and reward, respectively.

To integrate the constraints we proposed an improved autoencoder model named ImpAE, which combined Eqs. (6) and (7) then jointly minimizes the following loss function:

8 $$arg\mathop {\min }\limits_{\omega \in \Omega } \mathop \sum \limits_{i = 1}^{n} \left\| {y_{i} - x_{i} } \right\|_{2}^{2} + \gamma Loss_{c}$$

The loss function is constituted of two parts, the first part measures the squared error between output and input node features, the second part measures the error score of constraints in the hidden layer.

#### Disease similarity calculation

After obtaining low-dimensional feature representations of diseases by ImpAE, we calculated the disease similarity defined as the measurement of cosine distance of their feature vectors $W_{{d_{i} }} = \left\{ {W_{1,1} ,W_{1,2} , \ldots ,W_{1,i} , \ldots ,W_{1,N} } \right\}$ as following:

9 $$Sim\left( {d_{1} ,d_{2} } \right) = \frac{{\mathop \sum \nolimits_{i = 1}^{N} W_{1,i} \cdot W_{2,i} }}{{\sqrt {\mathop \sum \nolimits_{i = 1}^{N} W_{1,i}^{2} } \sqrt {\mathop \sum \nolimits_{j = 1}^{N} W_{2,j}^{2} } }}$$

#### The ImpAESim algorithm

The ImpAESim algorithm mainly contains two parts, a multi-network embedding algorithm for compact feature learning and a disease similarity calculation method based on distance measurement of feature vectors. In the compact feature learning part, we first ran the RWR process on each of the disease-related information network to learn the topological structure information, then we trained the original AE and ImpAE model to learn the low-dimensional representations of disease features. As the iterations increase, the model tends to be stable eventually. Then the cosine distance of disease feature vectors is computed as the disease similarity. The pseudocode for ImpAESim is shown in Algorithm 1.
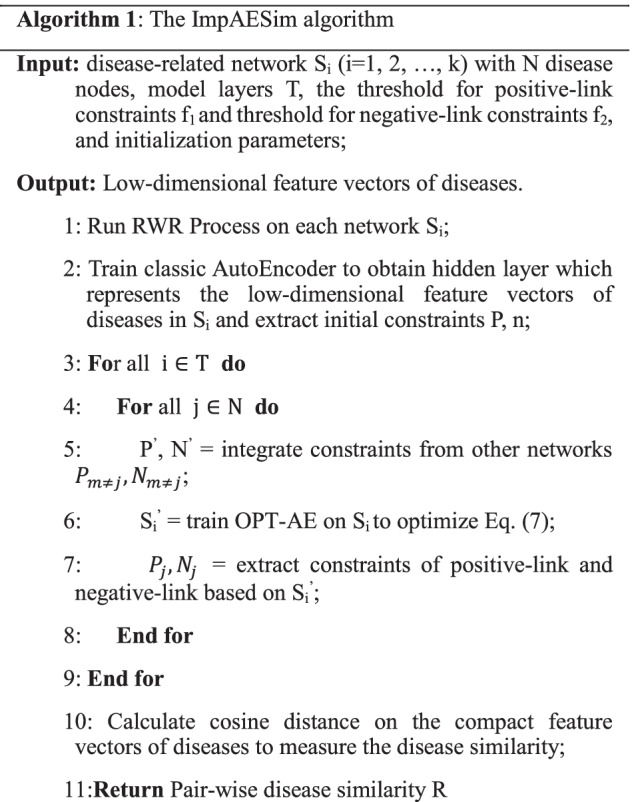


## Data Availability

All the datasets used in this paper could be downloaded from https://github.com/mymymaya/ImpAESim

## Abbreviations

AE: Auto encoder
RWR: Random walk with restart
SEMD: Spondyloepimetaphyseal dysplasia
PMDS: Persistent Mullerian duct syndrome
MODY: Maturity onset diabetes of the young
GSD: Glycogen storage disease
VSD: Ventricular septal defect
SMS: Smith–Magenis syndrome
DJS: Dubin–Johnson syndrome
ILL: Infantile liver failure
ADHD: Adrenocorticotropic hormone deficiency
HD: Hematological disease
FBCP: Familial benign chronic pemphigus
LEP: Leptin
SLC: Sideroblastic
SCDO: Spondylocostal dysostosis
CPHD: Combined pituitary hormone deficiency
CRMO: Chronic recurrent multifocal osteomyelitis
